# Intraocular pressure control efficacy and safety of HA-Mg glaucoma drainage plate implantation in the anterior chamber of rabbit eyes

**DOI:** 10.1007/s10856-024-06806-x

**Published:** 2024-06-25

**Authors:** Mingming Cai, Wangdu Luo, Kevin Feng, Yi Chen, Lin Yi, Xiaomin Zhu, Ju He, Hong Liu, Cindy Hutnik, Yong Wang, Xiangji Li, Lin Xie

**Affiliations:** 1 Department of Ophthalmology, The Third Affiliated Hospital of Chongqing Medical University, Chongqing, 401120 China; 2 Department of Ophthalmology, Beibei Hospital of Chongqing Medical University, Chongqing, 400700 China; 3 Department of Basic Medical Science, Western University, London, ON Canada; 4 School of Materials Science and Engineering, National Engineering and Technology Research Center for Magnesium Alloy Materials, Chongqing University, Chongqing, 400045 China; 5 Department of Ophthalmology, Ivey Eye Institute, St. Joseph’s Health Care London, Western University, London, ON Canada

## Abstract

**Abstract:**

The current clinical application of glaucoma drainage devices is made of non-degradable materials. These non-degradable drainage devices often trigger inflammatory responses and scar proliferation, possibly leading to surgical failure. We developed a biodegradable material hydroxyapatite-coated magnesium (HA-Mg) as a glaucoma drainage device. Twelve New Zealand white rabbits were randomly assigned to three groups: HA-Mg drainage plate group (6 right eyes), trabeculectomy group (6 right eyes), and control group (12 left eyes). Results showed that all HA-Mg drainage plates were completely degraded ~4 months postoperatively. At the 5th month postoperatively, there was no statistical difference in the corneal endothelium density between the HA-Mg drainage plate group and the control group (*p* = 0.857). The intraocular pressure (IOP) level in the HA-Mg drainage plate implantation group was lower than in the other two groups. The trypan blue dye still drained from the anterior chamber to the subconjunctiva 5 months after HA-Mg drainage plate implantation. HE staining revealed the scleral linear aqueous humor drainage channel and anterior synechia were observed after drainage plate completely degraded, with no obvious infiltration with the inflammatory cells. This study showed the safety and efficacy of HA-Mg glaucoma drainage plate in controlling IOP after implantation into the anterior chamber of rabbit eyes.

**Graphical abstract:**

**Figure:**
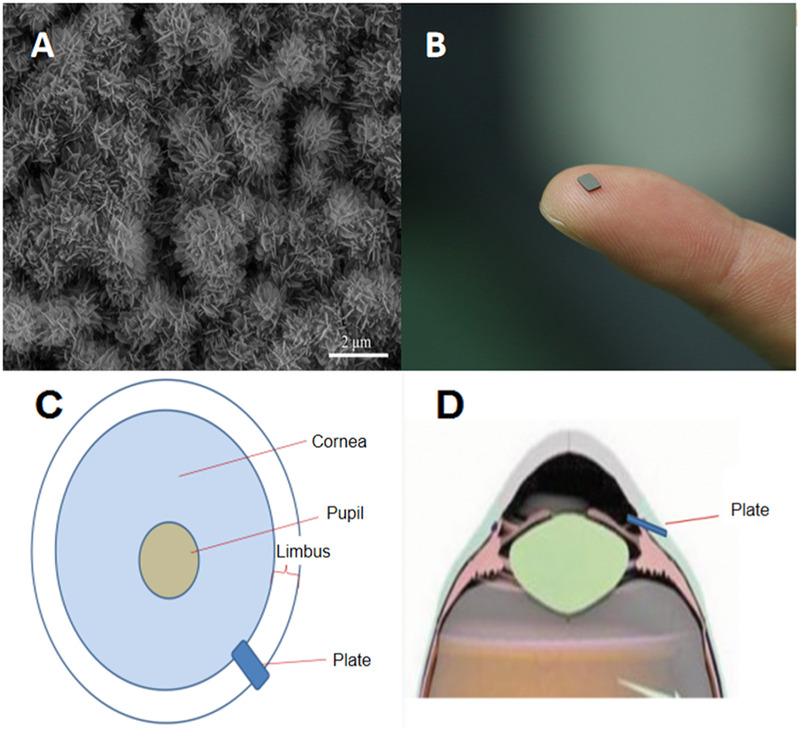
We developed a biodegradable material hydroxyapatite-coated magnesium (HA-Mg) as a glaucoma drainage device. This study evaluated the safety and efficacy of HA-Mg glaucoma drainage plate in controlling intraocular pressure after implantation into the anterior chamber of rabbit eyes.

## Introduction

Glaucoma comprises a group of characteristic optic nerve diseases marked by visual field defect and optic nerve atrophy. The World Health Organization classifies glaucoma as the second leading cause of high-risk blindness and the primary cause of irreversible blindness worldwide [1, 2]. Since 2010, there have been ~60.5 million patients diagnosed with primary open-angle glaucoma and primary angle-closure glaucoma globally. By 2020, this number reached ~79.6 million, and it is estimated to reach 111 million glaucoma patients worldwide by 2040 [3]. Subconjunctival drainage stands as the most foremost important surgical method and pathway for treating glaucoma [4]. In addition to trabeculectomy, various types of glaucoma drainage valves or devices have been widely used in clinical therapy, such as the Ahmed glaucoma valve implant, Ex-Press drainage implant, XEN, and Preserflo MicroShunt. However, the current clinical application of glaucoma drainage devices is made of non-degradable materials, such as silicone, stainless steel, titanium, gelatin, and cationic polymer materials. These non-degradable drainage devices, resembling foreign bodies beneath Tenon’s capsule, often trigger inflammatory responses, possibly acting as the primary risk factors for drainage channel blockages [5] and the formation of scar tissue around drainage devices [6–9]. On the contrary, due to the fixed diameter of the drainage tube, excessive aqueous humor drainage can occur during the early postoperative period, potentially leading to increased susceptibility to conditions like hypotony and a shallow anterior chamber [10]. Conversely, during the later postoperative, subconjunctival scarring, can impede the outflow of aqueous humor, causing an elevation in the intraocular pressure (IOP) and increasing the likelihood of surgical failure. Therefore, a great need exists for a more safety and efficacy microinvasive glaucoma surgery or microinvasive bleb surgery (MIBS).

In the preliminary study, we screened a hydroxyapatite-coated magnesium (HA-Mg) material through in vivo and in vitro experiments. This material had excellent biocompatibility, low cytotoxicity, and moderate anti-scarring effect [11]. Drawing from previous research findings and the surface structure characteristics of the hydroxyapatite (HA) coating, our research team developed a non-hollow tubular biodegradable glaucoma drainage plate. During the early postoperative phase, the tiny gaps on the coating surface facilitated aqueous humor drainage, while in the middle and later stages, continuous degradation of the drainage plate expanded the aqueous humor drainage channel increasing the volume of the aqueous humor. The objective of this study was to assess the safety and efficacy of a novel HA-Mg glaucoma drainage plate implanted into the anterior chamber of New Zealand rabbit eyes through an ab externo subconjunctival approach.

## Materials and methods

### Experimental animals

Twelve specific pathogen-free New Zealand rabbits, aged 3–4 months and weighing 1.8–2.0 kg each, were used in this study. The experimental animals were obtained from the Laboratory Animal Center of Chongqing Medical University. The research adhered to guidelines of the Chinese Animal Welfare Law and the ARVO statement concerning the use of animals in ophthalmic research. All animal experiments were conducted in accordance with the Regulations for the Administration of Laboratory Animals issued by the National Science Council of China. The 12 New Zealand rabbits were housed in standard cages (60 cm^2^), and they were fed with both clean water and a standard diet. The experimental animal license number is SYXK (Chongqing) 2022-0016. This study was approved by the Ethics Committee of the Third Affiliated Hospital of Chongqing Medical University, Ethics No. (2021) 14.

### Fabrication of HA-Mg glaucoma drainage plate

Magnesium, with a purity level of 99.99%, was sourced from the National Engineering Research Centre for Magnesium Alloys at Chongqing University, China. Through extrusion at a temperature of 270 °C, pure magnesium was transformed into a 9 mm magnesium rod. This rod was then machined into a rectangular drainage plate measuring 3 mm in height, 2 mm in width, and 0.3 mm in thickness. The four corners of the plate were chamfered, and its surface of the drainage plate was polished by 400#, 600#, 800#, and 1200# abrasive papers, respectively. Additionally, the pure magnesium drainage plate was used as the matrix, upon which the coating was applied through chemical deposition (HA, 2 μm in thickness). The samples of HA-Mg drainage plate were immersed in 70% ethanol for 10 min, washed twice with distilled water, and dried using a UV light.

**Fig. 1:**
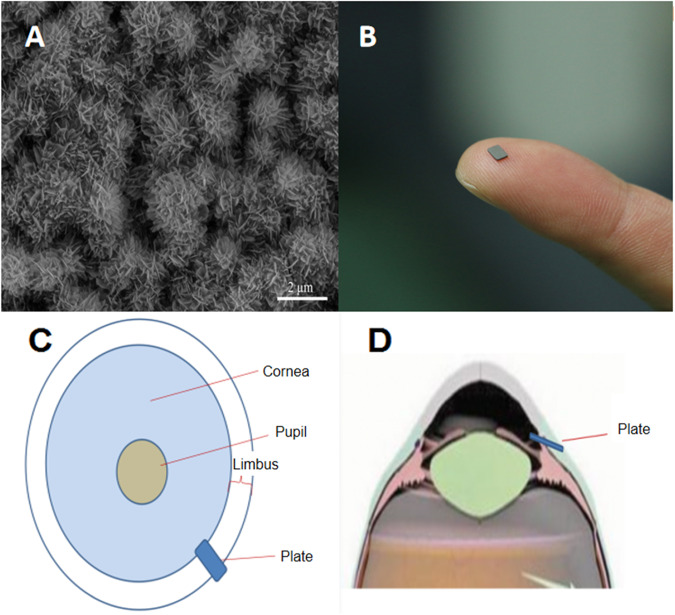
The gaps on hydroxyapatite (HA) coating surface and surgical approach of drainage plate. **A** Scanning electron microscope (SEM) images of HA coating surface morphology and drainage gaps at 20,000× magnification. **B** The appearance of HA-Mg glaucoma drainage plate. **C** The top view of the implanted plate in the eye. **D** The lateral view of the implanted plate

The gaps on the HA coating surface and the surgical approach of the drainage plate, as shown in Fig. 1, the electron microscope view highlighted the dense, crystal-like structure of the HA-coated surface alongside the gaps that were utilized for draining aqueous humor in the early postoperative period (Fig. 1A). The traditional drainage tube was modified into a non-hollow drainage plate (Fig. 1B). Instead, one end of the HA-Mg drainage plate was positioned within the anterior chamber, while the other end was placed under Tenon’s capsule (Fig. 1C). The drainage plate was fixed and limited in the scleral tunnel (Fig. 1D).

### Experimental design and grouping

Twelve New Zealand rabbits were randomly divided using a paired comparison method based on gender, age, and weight. To establish pairs, two essentially identical New Zealand rabbits were paired together, resulting in six pairs. Each pair of the New Zealand white rabbits was assigned as either number 1 or number 2. Subsequently, within each pair, the number 1 was coded as group A, while the number 2 was coded as group B, resulting in a total of 3 groups in total. The control group consists of 12 left eyes that did not undergo any intervention. In group A, the HA-Mg drainage plate was surgically implanted in the right eyes of the six rabbits. In group B, trabeculectomy was performed on the right eyes of six rabbits.

### Surgical procedure

#### Rabbit model of HA-Mg glaucoma drainage plate implantation

Rabbits were anesthetized using a 3% sodium pentobarbital injection administered into the vein of ear at a dosage of 1 ml/kg. For all cases, a fornix-based conjunctival flap was employed. A sclera tunnel was created using a 2.8 mm sclera tunnel and paracentesis knife, extending from 1.5 mm posterior of the limbus to the anterior chamber. The HA-Mg glaucoma drainage plate was implanted, ensuring that one end of the plate was positioned within the anterior chamber and the other end was situated under the Tenon’s capsule. The conjunctival flap was closed with an 8–0 vicryl suture. After completing the procedure, the rabbits were placed in an animal incubator set at 37 °C during the postoperative period. All eyes received tobramycin–dexamethasone administered twice daily.

#### Rabbit model of trabeculectomy

The rabbits were anaesthetized using a 3% sodium pentobarbital injection administered into the vein of ear at a dosage of 1 ml/kg prior to the operation. All subjects underwent a standard trabeculectomy procedure. A fornix-based conjunctival flap was employed for all cases. A partial thickness scleral flap was constructed and dissected anteriorly to the limbus, and extending into the clear cornea. The sclerotomy was performed through either sharp excision or by a punch, followed by a peripheral iridectomy. The scleral flap was closed with 10–0 nylon sutures, while the Tenon capsule and conjunctiva were closed with an 8–0 vicryl suture. After the completion of the surgical procedure, the rabbits were placed in an animal incubator set at 37 °C for postoperative care. All eyes received tobramycin–dexamethasone administered twice daily.

### Postoperative examination

During the 1st, 3rd, and 5th months following the surgery, the conjunctival filtering bleb, cornea, anterior chamber, lens, and anterior vitreous body were observed under a slit lamp microscope. The retina and optic nerve were observed under an indirect ophthalmoscope. Ultrasound biomicroscope (UBM) (SW-3200L, Suoer, China) was used to measure the fixation and displacement of the drainage plate.

### Corneal endothelium density measurement

The rabbits that were underwent HA-Mg glaucoma drainage plate implantation were selected at the 5th month after operation. The corneal endothelium density in both eyes (HA-Mg glaucoma drainage plate group, control group) was measured using a corneal endothelium density specular microscopy (EM-3000, TOMEY, Japan) after administering general anesthesia.

### Preoperative baseline IOP and postoperative IOP measurement

The IOP was measured daily using the Tonopen tonometer (Reichert, USA) during the week prior surgery to establish the baseline IOP. The changes in IOP following surgery were measured on a weekly basis. IOP measurements were taken after applying local anesthesia with oxybuprocaine drops (Santen, Japan). The Tonopen tonometer was positioned perpendicular to the center of the cornea, and each eye was measured for an average of three times.

### The patency of aqueous humor drainage channel test

At 1st, 3rd, and 5th months after operation, a rabbit from both the trabeculectomy group and the HA-Mg drainage plate implantation group was selected. After general anesthesia, 0.1 ml trypan blue dye (Surgi-Blue, Surgi Edge, India) was injected into the anterior chamber. The diffusion of trypan blue dye under the conjunctiva was observed 10 min after the injection. After each experiment, these two rabbits were not tested for patency of drainage channels again.

### Histology and immunofluorescent analysis

Rabbits were euthanized at the 6th month after surgery. The eyes were enucleated, fixed in 4% paraformaldehyde, and paraffin embedded. The samples were sectioned (4 μm in thickness) using the Microm HM550 (Carl Zeiss Ltd., Oberkochen, Germany). The sections were washed with xylene twice for 15 min each, then the slides were immersed in a series of ethanol solutions for 5 min each (twice in 100% (vol/vol), once in 95%, once in 80%, once in 70%), followed by 2-min exchanges in water. The sections were stained with hematoxylin and eosin (H&E) for histology analysis.

### Statistical analysis

The corneal endothelium density measurement results were analyzed using a paired-sample *T*-test. The IOP results were analyzed using a one-way ANOVA test. Statistical significance was accepted at *p* < 0.05. All statistical analyses were conducted using SPSS software (IBM Corp. Version 24.0. Armonk, NY, USA).

## Results

### Clinical examination and eye response

**Fig. 2:**
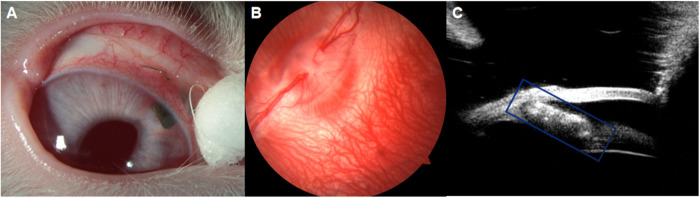
The anterior and posterior segments examination and the fixation of drainage plate. **A** The anterior segment of the eye 30 days post surgery. **B** The posterior segment of the eye 60 days post surgery. **C** The UBM image of the eye 30 days post surgery

A total of 12 New Zealand white rabbits were used in this study, with 6 rabbits undergoing trabeculectomy and the remaining 6 undergoing HA-Mg glaucoma drainage plate implantation. Following surgery, the rabbits displayed normal patterns of diet, drinking, and excretion. There were no instances of adverse effects, such as diarrhea, vomiting, lethargy, and appetite reduction. Physical observations indicated no congested conjunctiva, corneal opacification, or evident inflammation in the anterior chamber (Fig. 2A). The transparency of the lens and vitreous was maintained, and there were no indications of bleeding or pigmentation in the posterior segment of eye (Fig. 2B).

### The anterior and posterior segments examination and the fixation of drainage plate

All six HA-Mg drainage plates underwent complete biodegradation ~4 months after the operation. Among these, the UBM image revealed that six drainage plates were fixed well at 3 months after the operation (Fig. 2C). Four drainage plates were fixed well, while minimal rotation was noted in two drainage plates at 3 months after the operation. The two ends of these two drainage plates still remained situated in the anterior chamber and under Tenon’s capsule, respectively. No instances were observed of drainage plates detaching into the anterior chamber.

### Corneal endothelium density

**Fig. 3:**
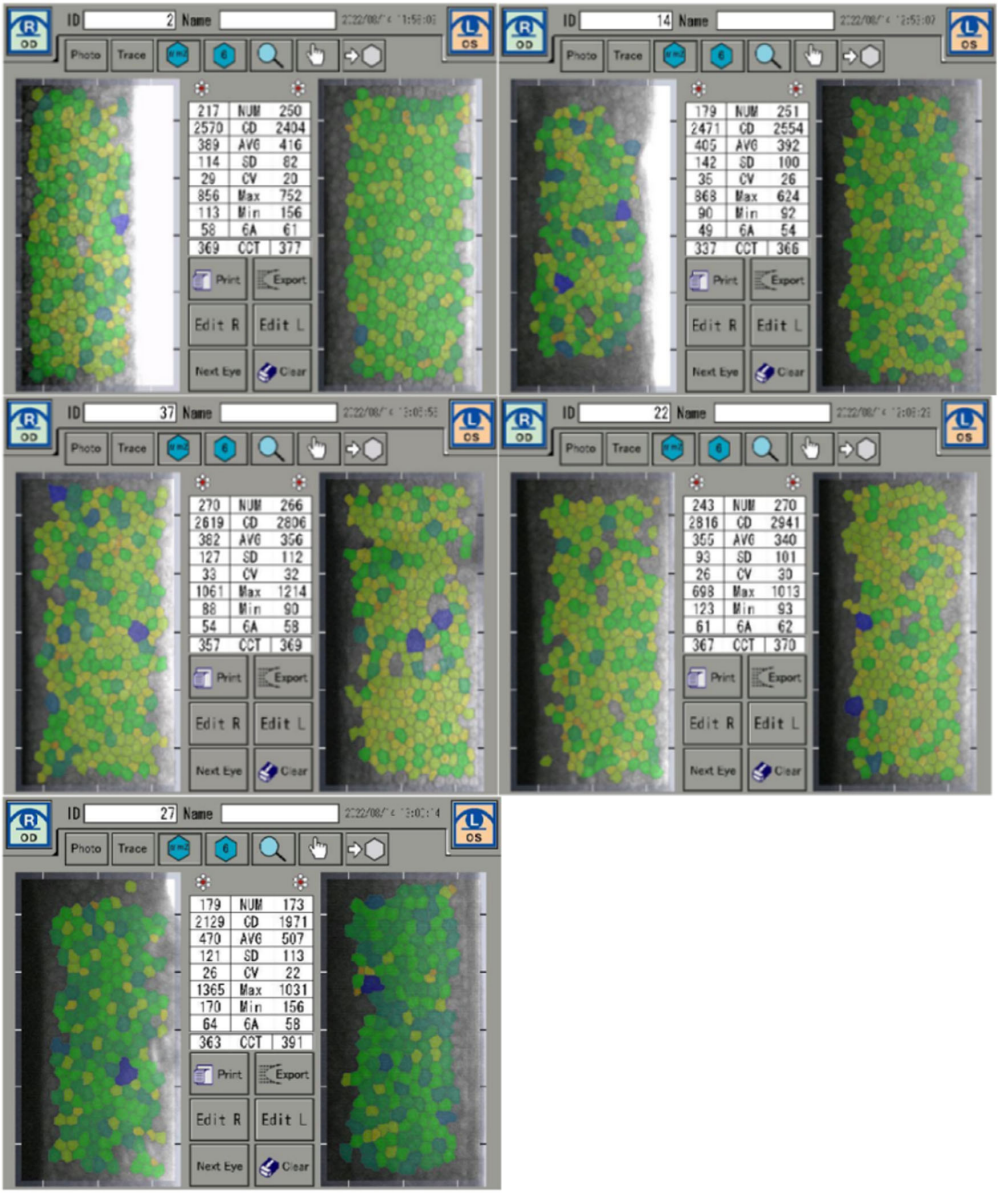
The corneal endothelium density measurement. Five rabbits corneal endothelium density specular microscopy examination reports, OD: HA-Mg drainage plate group. OS: control group

**Fig. 4:**
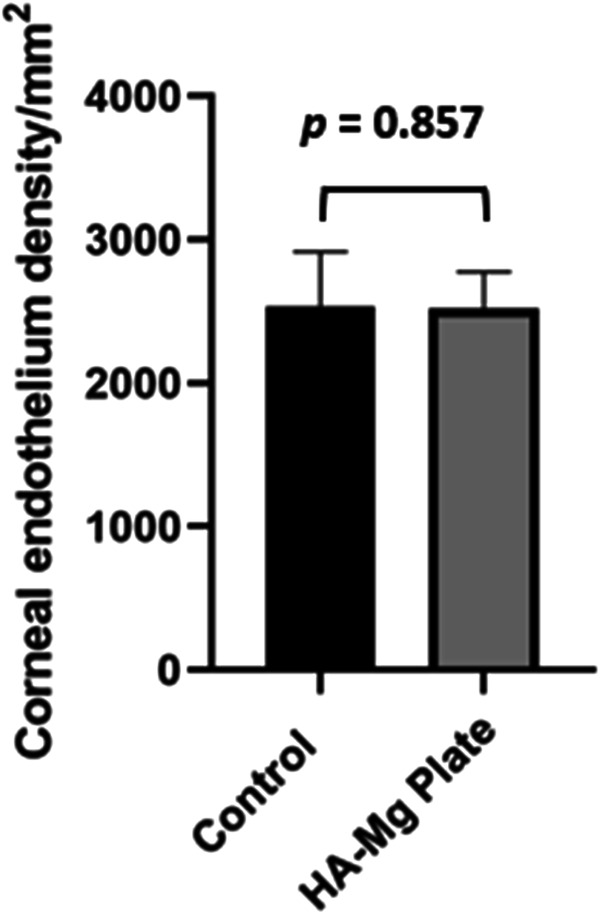
The density of corneal endothelium. The *p* value of the different groups was no statistical difference (*p* = 0.857). Data were analyzed using a paired-sample *T*-test, *N* = 5

Corneal endothelium density measurements were performed at 5th month following surgery. Five rabbits corneal endothelium density specular microscopy examination reports shown in Fig. 3, the density and morphology of corneal endothelial cells within the HA-Mg drainage plate group (OD) exhibited similarities to those in the contralateral eye (OS). A paired-sample *T*-test was employed to compare the corneal endothelium between the two groups (*P* = 0.857) revealing no statistically significant difference (Fig. 4).

### Preoperative baseline IOP and postoperative IOP measurement

The IOP of rabbit eyes was measured using a Tonopen tonometer on a daily basis during the leading up to the experiment. The baseline IOP ranged between 12 and 15 mmHg, additionally, the difference of IOP between two eyes was <5 mmHg.

Throughout the continuous IOP monitoring spanning 21 weeks, the outcomes are shown in Table 1. To assess the weekly changes in IOP across the three groups, a one-way ANOVA analysis was conducted. Analysis revealed that there were statistically significant differences in IOP changes among the groups at different time points (*p* < 0.05). In the first 5 weeks post-operation, both the HA-Mg drainage plate group and trabeculectomy group displayed lower IOP levels than control group. Subsequently, from the 6th week onwards, the IOP of the trabeculectomy group reverted to a normal range. Following surgery, the IOP levels of the HA-Mg drainage plate group remained lower than the trabeculectomy group and the normal control. A further reduction of IOP was observed from 15th to 16th week post surgery (Table 2).

**Table 1:** Experimental and group design

Serial number	Right eye	Left eye
A1	HA-Mg drainage plate	Control
A2	HA-Mg drainage plate	Control
A3	HA-Mg drainage plate	Control
A4	HA-Mg drainage plate	Control
A5	HA-Mg drainage plate	Control
A6	HA-Mg drainage plate	Control
B1	Trabeculectomy	Control
B2	Trabeculectomy	Control
B3	Trabeculectomy	Control
B4	Trabeculectomy	Control
B5	Trabeculectomy	Control
B6	Trabeculectomy	Control

Twelve New Zealand rabbits in respective groups

### The surgery procedure and patency of aqueous humor drainage channel test

**Fig. 5:**
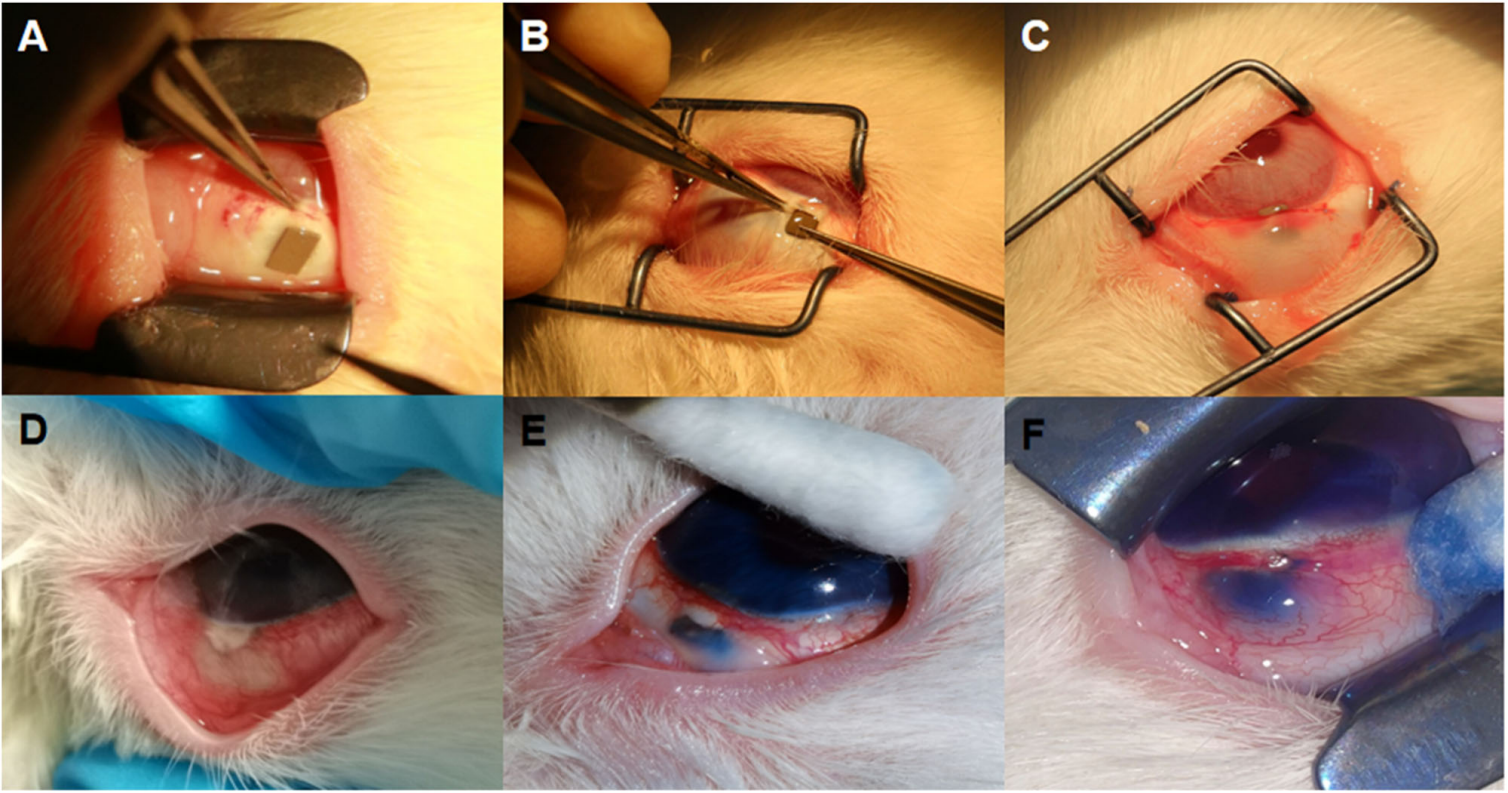
Surgery procedure and postoperative drainage channel measurement. **A**–**C** Surgery procedure of HA-Mg glaucoma drainage plate implantation. **A** The appearance of HA-Mg glaucoma drainage plate and sclera tunnel. **B** HA-Mg glaucoma drainage plate implanted. **C** The appearance after surgery. **D**–**F** Postoperative drainage channel measurement. **D** 1st month after trabeculectomy. **E** 1st month after HA-Mg glaucoma drainage plate implantation. **F** 5th month after HA-Mg glaucoma drainage plate implantation

As shown in Fig. 5, the surgery procedure of HA-Mg glaucoma drainage plate implantation went very smooth (Fig. 5A–C), the drainage plate was fixed well (Fig. 5B), and the conjunctival filtering bleb formed well after surgery (Fig. 5C). At the 1st month post surgery, the injection of trypan blue dye into the anterior chamber resulted in the absence of any observed filtration of the dye from the anterior chamber to the subconjunctiva within the trabeculectomy group (Fig. 5D). Moreover, from the 1st month following the operation, none of the rabbits that underwent trabeculectomy exhibited the ability to filter trypan blue dye to the subconjunctiva during the aqueous humor drainage channel patency test. On the other contrary, within the HA-Mg drainage plate group at the 1st month post surgery, the drainage plate’s two ends were located in the anterior chamber and subconjunctiva, respectively, the trypan blue dye was observed to drain into the subconjunctiva space (Fig. 5E). By 5th month post surgery, complete degradation of the HA-Mg drainage plate had taken place, and the trypan blue dye was still drained out under the conjunctival filtering bubble (Fig. 5F) after being injected into the anterior chamber.

**Table 2:** Results of continuous weekly IOP measurements post surgery. (Week, mmHg)

Week	HA-Mg plate group	Trabeculectomy group	Control group	*F* value	*P* value
1st	9.33 ± 1.92	10.67 ± 2.21	13.33 ± 1.416	11.635	0.000
2nd	11.00 ± 0.94	11.16 ± 0.62	14.00 ± 0.96	32.689	0.000
3rd	12.55 ± 0.71	12.33 ± 0.60	14.25 ± 0.75	19.502	0.000
4th	11.00 ± 0.79	12.50 ± 0.35	13.00 ± 1.02	11.228	0.000
5th	11.67 ± 0.42	12.33 ± 0.30	13.41 ± 0.51	33.091	0.000
6th	11.16 ± 0.50	12.66 ± 0.79	12.42 ± 0.68	9.108	0.001
7th	12.16 ± 0.86	13.00 ± 0.99	13.42 ± 0.73	4.567	0.023
8th	11.67 ± 0.37	14.00 ± 0.59	13.75 ± 0.43	50.122	0.000
9th	12.67 ± 0.56	14.67 ± 0.73	14.75 ± 0.35	35.772	0.000
10th	11.50 ± 0.28	14.17 ± 0.28	14.42 ± 0.18	136.041	0.000
11th	11.83 ± 0.55	14.33 ± 0.21	14.16 ± 0.50	60.299	0.000
12th	12.17 ± 0.18	14.33 ± 0.63	13.83 ± 0.61	27.027	0.000
13th	11.83 ± 0.75	13.16 ± 1.05	13.00 ± 0.43	6.962	0.005
14th	11.00 ± 0.47	13.50 ± 0.69	13.33 ± 0.57	38.495	0.000
15th	10.00 ± 0.56	13.00 ± 0.47	12.66 ± 0.47	61.766	0.000
16th	9.66 ± 0.47	13.66 ± 0.92	12.83 ± 0.52	70.982	0.000
17th	9.33 ± 0.30	13.60 ± 0.43	13.54 ± 0.64	140.881	0.000
18th	9.60 ± 0.28	13.80 ± 0.60	13.30 ± 0.62	92.953	0.000
19th	10.00 ± 0.53	13.60 ± 0.36	13.50 ± 0.42	122.755	0.000
20th	9.80 ± 0.45	13.40 ± 0.28	13.30 ± 0.33	191.601	0.000
21st	10.00 ± 0.24	13.60 ± 0.55	12.80 ± 0.50	86.256	0.000

### Histology of aqueous humor drainage channel

**Fig. 6:**
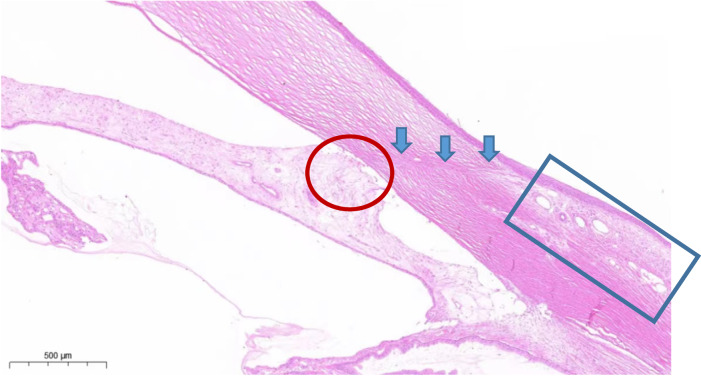
Hematoxylin and eosin staining of HA-Mg glaucoma drainage plate implantation. Blue arrow: aqueous humor drainage channel. Red circle: anterior synechia. Blue rectangle: raised, microcystic filtering bubble

Representative H&E staining images of the specimens are shown in Fig. 6. Within the HA-Mg glaucoma drainage plate, complete degradation was evident. This allowed for the construction and persistence of a linear physiological aqueous drainage channel. Consequently, the development of a functional filtering bubble was formed, characterized by a looser and microcystic-like tissues, whereas the anterior synechia could be observed. The inflammatory response was not intense across all tissues.

## Discussion

The success rate of glaucoma drainage device implantation is influenced by several key risk factors. In the case of distal outflow, bleb-forming devices, these include the obstruction of aqueous humor egress due to inflammation-driven excessive fibrosis often leading to encapsulation of the drainage device or blockage of the devices outflow channels [12–14]. Glaucoma drainage tube or devices, which have one end located in the anterior chamber for an extended period, carry the risk of continuous friction with the corneal endothelium. This friction can result in corneal endothelium damage and corneal endothelium decompensation. Large devices that are positioned in the subconjunctival space and their connecting tubes can lead to erosion and site-threatening endophthalmitis. These complications can require the devices to be removed, leading to failure of the glaucoma surgery and, in some cases, the requirement for other surgeries such as endothelial keratoplasty [15–17]. For example, the CyPass Micro-Stent implant, due to the strong scar healing reaction of polyamide material, the stent is prone to be encapsulated by fibers, leading to early postoperative failure of the stent implantation. More seriously, 5 years of clinical follow-up observation revealed a continuous corneal endothelium damage after surgery [18–20]. As a result, a global recall of this product was announced in August 2018.

Compared to other medical implant materials, in addition to excellent biocompatibility, the most significant advantage of magnesium-based materials is that they can be degraded in the physiological environment with no toxic side effects [21–23]. Given these advantages, ophthalmologists began to use pure magnesium materials for surgical intervention targeting glaucoma treatment in the mid-20th century [24, 25]. However, its application potential was limited by the rapid and non-uniform degradation of pure magnesium in the eye, as well as the large amount of hydrogen released during the degradation process. Currently, with the development and maturity of surface modification techniques, the defects of magnesium-based materials corrosion have been affectively addressed. HA, known for its excellent biocompatibility and its similarity to human bone tissue, is commonly used for intraorbital implants following eyeball removal. It is an essential carrier for artificial eyeballs [26, 27]. Furthermore, HA holds significant promise in artificial cornea application [28, 29]. In this study, we expected the HA-Mg glaucoma drainage plate may be able to create a physiological channel once degraded is different from currently available MIBS devices.

The current study examined both the in vivo safety and efficacy of the HA-Mg glaucoma drainage plate. Twelve New Zealand white rabbits underwent surgical implantation with several parameters monitored postoperatively. The rabbits exhibited normal diet, drinking, and excretion patterns. They did not exhibit any adverse effects, such as diarrhea, vomiting, lethargy, or appetite reduction. There were no signs of congested conjunctiva, corneal opacification, or apparent inflammation in the anterior chamber. The transparency of the lens and vitreous was preserved and no bleeding or pigmentation was observed in the posterior segment of eye. Neither systemic nor ocular complications were observed, confirming the biosafety of the HA-Mg glaucoma drainage plate was well and verified. The study also carefully assessed the stability and immobility of the plate as no limiting or fixed part was incorporated into this drainage plate design. The lack of migration was likely due to the crystal-like structure on the surface of HA coating, which has a certain friction level without inciting significant fibrosis. Moreover, the ~2 mm scleral tunnel is expected to effectively limit and fixing the drainage plate. The result showed that four drainage plates were securely fixed, while a minimal rotation was observed for two drainage plates at 3rd month after surgery. Both ends of the drainage plates were located in the anterior chamber and subconjunctiva, respectively. The rotational movement of the drainage plate was related to the slight horizontal expansion of the incision, caused by the 2.8 mm sclera tunnel knife during the challenging drainage plate implanting. None of the drainage plates dislocated into the anterior chamber. The fixation and stability performance of the HA-Mg drainage plate was encouraging and supportive of further investigation for refinement in future studies. The biodegradable composition of the HA-Mg glaucoma drainage plate, its size, and location all were demonstratable favorable features to reduce the the risk of long-term friction with the corneal endothelium. However, what remains to be determined are future studies specifically designed to examine longer term effects of the material degradation and its products on the corneal endothelium. The lack of any significant inflammatory response was reassuring. The results showed that all HA-Mg drainage plates were completely degraded 4 months after surgery, with the corneal endothelium density measurements performed at 5th months post surgery. There was no significant difference in corneal endothelium morphology or density between the HA-Mg drainage plate implanted eyes and the contralateral eyes (control group, without surgery). After implantation of the HA-Mg glaucoma drainage plate in the New Zealand white rabbit eyes, the procedure showed excellent biosafety, was well fixed in the anterior chamber, and the degradation of the material did not result in corneal endothelium damage or decline.

The challenges in currently available devices were outlined earlier. Our hypothesis is that a drainage device can be designed to improve the safety and efficacy of glaucoma surgery. In the early postoperative period, the gaps within the HA coating can be attributed to the successive outflow of humor aqueous. This controlled drainage minimizes the volume of drained aqueous humor, reducing the decrease in IOP, and lowering the risk of ocular hypotony complications. Conversely, during the later period, an increase in the outflow of aqueous humor and a larger volume of aqueous humor is drained to the subconjunctiva improving surgical success rate.

This study found that, in the trabeculectomy group, the IOP returned to a pre-surgical IOP level around the 6th week, which indicates that the aqueous humor filtration channel in rabbit eyes likely healed and became obstructed around 5 weeks after trabeculectomy, even without the intraoperative drug application and postoperative eye massage. The IOP level of the HA-Mg drainage plate group was lower than the trabeculectomy group and the control group after surgery, and further reduction of IOP was observed between 15 and 16 weeks post surgery. We speculated that as the drainage plate undergoes continuous degradation, the aqueous humor drainage channel may gradually expand without undergoing adhesion healing, leading to the increased outflow of aqueous humor. The contact of the plate’s surface with the sclera tissue moderates fibroblast proliferation due to mild corrosion of HA-Mg degradation [11], effectively preventing subconjunctival scarring once the drainage plate is fully degraded. Notably, the postoperative scar formation typically takes more than 2 weeks, usually extending around 15 weeks [30]. This process seems to coincide with the degradation cycle of HA-Mg drainage plate. Therefore, scars can form around the HA-Mg implant during the degradation process, and an unblocked channel will be constructed after the full degradation due to the termination of scarring. On the other hand, the drainage channel patency test showed that while the trypan blue dye could not be filtered from the anterior chamber to the subconjunctiva at 1 month after trabeculectomy, the trypan blue dye could still be drained to the subconjunctiva at 5 months after the implantation of HA-Mg glaucoma drainage plate, even though the HA-Mg drainage plate had been completely degraded. The drainage channel patency test results corresponded closely with the weekly IOP measurements. The eye tissue section and HE staining outcomes at 6th month post surgery further indicated that after the complete degradation of HA-Mg drainage plate, the aqueous humor drainage channel was still visible. This was evident as the filtering bubble was raised and microcystic-like tissue was formed. Additionally, the anterior synechia further proved that the aqueous humor drainage channel was present.

However, there are many limitations in this study. First, the primary aim of this study was to examine the safety of a novel HA-Mg drainage plate in New Zealand white rabbit eyes. The secondary aim was IOP lowering efficacy. In the absence of a glaucoma model, it is uncertain if elevated pre-surgical IOPs will impact the experimental outcomes. We plan to establish a glaucoma model in the later experiments to further verify our findings. Second, the sample size was relatively small, and two experimental animals in the HA-Mg drainage plate group died at 4th month and 5th month after surgery. Although analysis found that there was no causal relationship with the implantation of drainage plate, it may still have some impact on the experimental results. Additionally, the observation time of this study was limited, and the potential reasons and mechanisms of aqueous humor drainage channel construction and maintenance during the degradation of drainage plate remain unclear. The long-term efficacy of the aqueous humor drainage channel after the complete degradation of drainage plate remains to be determined. Further research into these mechanisms is necessary for exploration and confirmation. Finally, the design of the HA-Mg glaucoma drainage plates in this study was a prototype. The minimal rotation observed in two drainage plates and the anterior synechia all indicate that further design and research is warranted.

## Conclusions

In this study, no significant systemic and intraocular side effects were observed after implantation of the HA-Mg glaucoma drainage plate in rabbit eyes, and no corneal endothelium effects were observed. The HA-Mg drainage plate implantation decreased IOP in rabbit eyes for an extended period when compared with trabeculectomy. The degradation of the drainage plate led to the formation of an unobstructed scleral drainage channel for aqueous humor. This study showed the safety and efficacy of HA-Mg glaucoma drainage plate in controlling IOP after implantation into the anterior chamber of rabbit eyes. It is believed that in the near future, new drainage devices with biodegradable materials will apply to enhance safety and efficacy in glaucoma treatment.
